# Dynamic Contrast-Enhanced MRI of Prostate Lesions of Simultaneous [^68^Ga]Ga-PSMA-11 PET/MRI: Comparison between Intraprostatic Lesions and Correlation between Perfusion Parameters

**DOI:** 10.3390/cancers13061404

**Published:** 2021-03-19

**Authors:** Jing Zhao, Avan Kader, Dilyana B. Mangarova, Julia Brangsch, Winfried Brenner, Bernd Hamm, Marcus R. Makowski

**Affiliations:** 1 Institute of Radiology and Nuclear Medicine, Charité—Universitätsmedizin Berlin, Corporate Member of Freie Universität Berlin, Humboldt-Universität zu Berlin, and Berlin Institute of Health, Charitéplatz 1, 10117 Berlin, Germany; avan.kader@charite.de (A.K.); dilyana.mangarova@charite.de (D.B.M.); julia.brangsch@charite.de (J.B.); bernd.hamm@charite.de (B.H.); marcus.makowski@charite.de (M.R.M.); 2 Department of Biology, Chemistry and Pharmacy, Institute of Biology, Freie Universität Berlin, Königin-Luise-Str. 1-3, 14195 Berlin, Germany; 3 Department of Veterinary Medicine, Institute of Veterinary Pathology, Freie Universität Berlin, Robert-von-Ostertag-Str. 15, Building 12, 14163 Berlin, Germany; 4 Department of Nuclear Medicine, Charité—Universitätsmedizin Berlin, Corporate Member of Freie Universität Berlin, Humboldt-Universität zu Berlin, and Berlin Institute of Health, Augustenburger Platz 1, 13353 Berlin, Germany; winfried.brenner@charite.de; 5 Department of Diagnostic and Interventional Radiology, Klinikum rechts der Isar, Technische Universität München, Ismaninger Str. 22, 81675 Munich, Germany

**Keywords:** prostate cancer, PSMA, PET/MRI, dynamic contrast-enhanced, arrival time, time to peak, wash-in slope, wash-out slope, peak enhancement intensity, initial area under curve

## Abstract

**Simple Summary:**

Dynamic contrast-enhanced magnetic resonance imaging (DCE-MRI) is an important method to analyze the perfusion model of tumors, allowing noninvasive quantification of microvascular structure and function. Furthermore, simultaneous [^68^Ga]Ga-prostate-specific membrane antigen (PSMA)-11 positron emission tomography (PET)/MRI is currently the most advantageous way for assessing prostate cancer staging. Therefore, combining these two examinations helps to diagnose the lesions more comprehensively. Our study analyzes perfusion parameters between intraprostatic lesions and the correlation between perfusion parameters and [^68^Ga]Ga-PSMA-11 PET. This study highlights the significant effect of PSMA uptake on perfusion parameters.

**Abstract:**

We aimed to retrospectively compare the perfusion parameters measured from dynamic contrast-enhanced (DCE) magnetic resonance imaging (MRI) of prostate benign lesions and malignant lesions to determine the relationship between perfusion parameters. DCE-MRI was performed in patients with PCa who underwent simultaneous [^68^Ga]Ga-prostate-specific membrane antigen (PSMA)-11 positron emission tomography (PET)/MRI. Six perfusion parameters (arrival time (AT), time to peak (TTP), wash-in slope (W-in), wash-out slope (W-out), peak enhancement intensity (PEI), and initial area under the 60-s curve (iAUC)), and a semi-quantitative parameter, standardized uptake values maximum (SUVmax) were calculated by placing regions of interest in the largest area of the lesions. The DCE-MRI parameters between prostate benign and malignant lesions were compared. The DCE-MRI parameters in both the benign and malignant lesions subgroup with SUVmax ≤ 3.0 and SUVmax > 3.0 were compared. The correlation of DCE-MRI parameters was investigated. Malignant lesions demonstrated significantly shorter TTP and higher SUVmax than did benign lesions. In the benign and malignant lesions subgroup, perfusion parameters of lesions with SUVmax ≤ 3.0 show no significant difference to those with SUVmax > 3.0. DCE-MRI perfusion parameters show a close correlation with each other. DCE-MRI parameters reflect the perfusion characteristics of intraprostatic lesions with malignant lesions, demonstrating significantly shorter TTP. There is a moderate to strong correlation between DCE-MRI parameters. Semi-quantitative analysis reflects that malignant lesions show a significantly higher SUVmax than benign lesions.

## 1. Introduction

Prostate cancer (PCa) is one of the most common diseases in men. In the US, it accounts for 21% of new cases, and it is the second leading cause of death in men, accounting for 10% of all deaths [1]. 

Multiparameters magnetic resonance imaging (mpMRI) with the assessment of images using the Prostate Imaging Reporting and Data System (PI-RADS) is widely used to evaluate prostate lesions. Dynamic contrast-enhanced (DCE)-MRI is a technique used to measure the perfusion, blood flow, and tissue vascularity by analyzing the tissue’s signal enhancement curve. DCE-MRI, which can assess micro-vascular properties, provides helpful additional information for characterizing lesions [2]. In many current clinical trials, DCE-MRI for a new anti-angiogenic agent is used as an early imaging biomarker to evaluate patients’ response to treatment [3]. Microvessel density has been reported to be associated with tumor stage, recurrence, metastatic potential, and prognosis in patients with prostate cancer [4,5,6]. This signal enhancement of perfusion can be quantified with semi-quantitative analysis [7,8]. Semi-quantitative parameters can be extracted and calculated from the signal intensity curve [9,10]. The signal intensity curve reveals several parameters, including arrival time (AT), time to peak (TTP), wash-in slope (W-in), wash-out slope (W-out), peak enhancement intensity (PEI), and initial area under the 60-sec curve (iAUC). AT is arrival time, which is the time point when contrast enhancement starts. TTP is time to peak, which is the time from arrival time to the end of wash-in. A shorter TTP indicates the shorter time needed to reach the peak. Wash-in slope is the slope of the fitted line between AT and the end of wash-in. The higher W-in is, the faster the wash-in speed. W-out slope is the fitted line slope between the start of wash-out and the end of the measurement. The higher the W-out is, the faster the wash-out speed. PEI indicates the highest value of enhancement. And iAUC calculates the initial area under the curve in 60 s, reflecting the total intensity of enhancement during the first one minute. 

Molecular imaging of prostate cancer is a beneficial tool for systematically evaluating tumor biology [11]. Agents targeting cell metabolism, hormone receptors, or membrane proteins have been developed to an advanced stage. Over the last few years, prostate-specific membrane antigen (PSMA) have gained much interest as specific targets for PCa imaging, which is a promising and specific target. It is a transmembrane glycoprotein related to tumor progression and disease recurrence that has been found to be overexpressed in prostate cancer cells [12,13]. PSMA-ligands as prostate cancer-specific PET tracers show and differentiate cancerous lesions within the prostate more accurately than other tracers. A whole-body hybrid PET/MRI scanner with simultaneous acquisition of PET imaging and mpMRI has enabled functional and molecular information to be combined [14,15,16]. Initial results suggest that [^68^Ga]Ga-PSMA-11 PET/MRI is a beneficial imaging method for detecting suspicious focal prostate cancer lesions [17,18] and monitoring recurrence [19]. Combining MRI and positron emission tomography (PET) improves diagnostic accuracy [20,21]. Compared with the current standard imaging like CT, MRI, and bone scintigraphy, PSMA-PET imaging shows a higher specificity and sensitivity and is suitable for patients with primary middle-risk or high-risk prostate cancer [22].

Therefore, this study’s purpose was to retrospectively compare the perfusion parameters of DCE-MRI of prostate benign lesions and malignant lesions, and determine the correlation between these perfusion parameters.

## 2. Results

### 2.1. Comparison of Parameters between Benign and Malignant Lesions

TTP and SUVmax were significantly different between benign lesions and malignant lesions (*p* < 0.05) regarding the perfusion parameters and semi-quantitative parameters. No significant differences were observed in other parameters (Table 1).

### 2.2. Effect of SUVmax on DCE-MRI Parameters

All lesions were assigned into two subgroups, including benign lesions group and malignant lesions group. In both subgroups, DCE-MRI parameters between lesions with SUVmax ≤ 3.0 and SUVmax > 3.0 were compared. In the benign lesions subgroup, perfusion parameters of lesions with SUVmax ≤ 3.0 show no significant difference from those with SUVmax > 3.0 (Table 2). In the malignant lesions subgroup, perfusion parameters of lesions with SUVmax > 3.0 perfusion parameters of lesions with SUVmax ≤ 3.0 show no significant difference from those with SUVmax > 3.0 (Table 3).

### 2.3. Pearson Correlation between Perfusion Parameters of Intraprostatic Lesions

There is a moderate to strong correlation between the perfusion parameters of intraprostatic lesions (Table 4).

## 3. Discussion

DCE-MRI is an important diagnostic method in detecting focal prostate cancer lesions, which improves the accuracy of examination for detection and evaluation of intraprostatic tumor lesions [23]. It visualizes focal lesions in the prostate with varying degrees of enhancement and provides information for lesion characterization. Combining the advantages of [^68^Ga]Ga-PSMA-11 PET/MRI and DCE-MRI contributes to a better differentiation of intraprostatic lesions [24]. PSMA-PET imaging can add molecular information to multiparameter MRI to describe suspicious lesions for target biopsy [25]. The clinical value of this study is the quantitative analysis of the multimodality characteristics of the lesions. DCE parameters reflect lesions’ microvascular structure, while SUVmax reflects lesions’ prostate-specific membrane antigen concentration. A combination of information allows for a comprehensive evaluation of tumor condition and for choosing an appropriate treatment plan.

**Figure 1:**
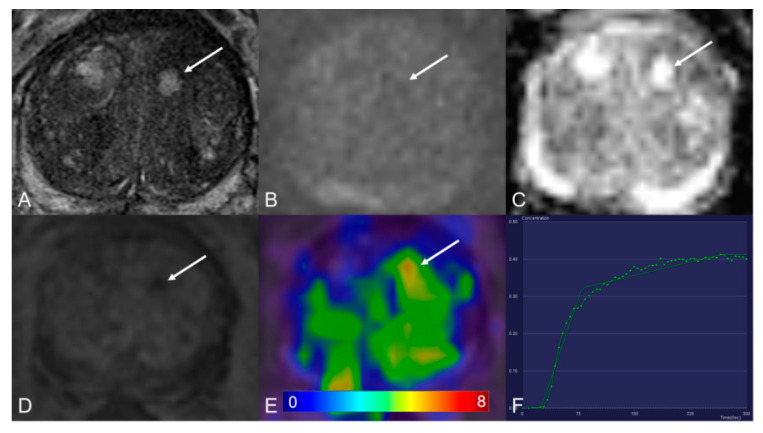
Transition zone with a PI-RADS 1 change. (**A**) Axial T2WI shows typical benign prostatic hyperplasia (BPH) change. (**B**) DWI (b = 1000 s/mm^2^) shows no lesion with a marked hyperintense signal above the background. (**C**) ADC map image presents no diffusion restriction. (**D**) Early dynamic contrast-enhanced image presents no enhancement within the typical BPH nodule. (**E**) [^68^Ga]Ga-PSMA-11 PET/MRI fusion image shows moderate [^68^Ga]Ga-PSMA-11 uptake, with SUVmax of 6.3. (**F**) DCE-MRI time-intensity curve demonstrates persistent increase enhancement. AT: 0.39 min; TTP: 1.09 min; W-in: 0.16; W-out: 0.02; PEI: 0.25; iAUC: 0.10.

**Figure 2:**
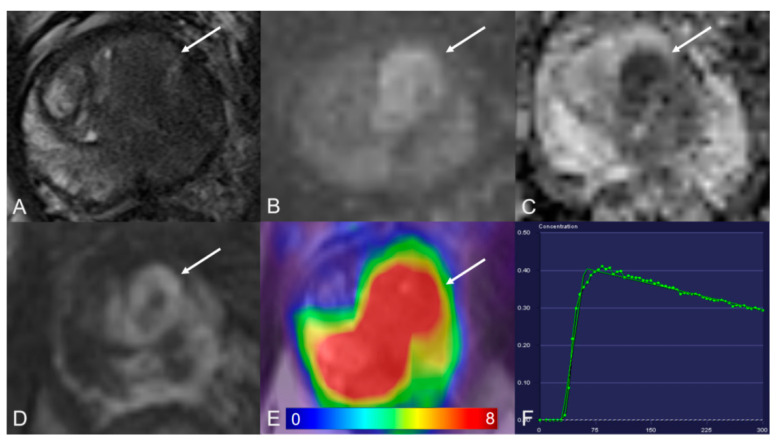
Transition zone with a PI-RADS 5 lesion. (**A**) Axial T2WI shows homogeneous hypointense. (**B**) DWI (b = 1000 s/mm^2^) shows a marked hyperintense signal above the background. (**C**) ADC map image presents a lesion with hypointense signal below the background. (**D**) Early dynamic contrast-enhanced image presents positive enhancement within the lesion. (**E**) [^68^Ga]Ga-PSMA-11 PET/MRI fusion image shows avid [^68^Ga]Ga-PSMA-11 uptake, with SUVmax of 29.2. (**F**) DCE-MRI time-intensity curve demonstrates a decline after initial up-slope enhancement. AT: 0.47 min; TTP: 0.50 min; W-in: 0.80; W-out: −0.03; PEI: 0.41; iAUC: 0.31.

**Figure 3:**
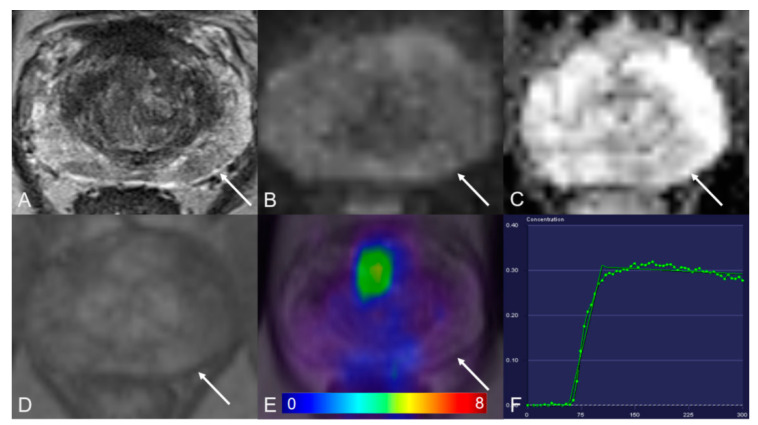
Peripheral zone with a PI-RADS 3 lesion. (**A**) Axial T2WI shows hypointense. (**B**) DWI (b = 1000 s/mm^2^) shows a mild hyperintense signal. (**C**) ADC map image presents a moderate hypointense signal below the background. (**D**) Early dynamic contrast-enhanced image presents no early enhancement within the lesion. (**E**) [^68^Ga]Ga-PSMA-11 PET/MRI fusion image shows no positive [^68^Ga]Ga-PSMA-11 uptake. (**F**) DCE-MRI time-intensity curve demonstrates plateau enhancement. AT: 0.98 min; TTP: 0.67 min; W-in: 0.26; W-out: 0.003; PEI: 0.20; iAUC: 0.13.

Intraprostatic lesions perfusion parameters are investigated by several studies before [26]. Vos et al. [27] reported that quantitative parameters and semi-quantitative parameters derived from DCE-MRI at 3.0 T MRI could assess the aggressiveness of PCa in the peripheral zone. Chen et al. [28] proved that the wash-out gradient shows a significant association with Gleason score and good diagnostic performance in assessing prostate tumor aggressiveness. PCa has increased microvascularity and, therefore, can be detected by contrast-enhanced MRI techniques, as van Niekerk et al. reported [29]. These parameters provide detailed information about the aggressiveness of tumors in different prostate gland regions as, for example, in Figure 1, Figure 2 and Figure 3. Therefore, the perfusion differences between prostate benign lesions and malignant lesions may be detected and quantified with DCE-MRI. MpMRI of prostate scanning includes complementary and synergistic T2, diffusion, and perfusion sequences. Ren et al. [30] proved that DCE-MRI curves could differentiate benign tissue from malignant prostate tissue based on T2-weighted imaging. The omission of DCE-MRI increases the risk that some aggressive lesions will not be detected, thus discrediting prostate imaging by MRI. The sequence of contrast enhancement agents is essential in the detection of recurrence and post-treatment follow-up.

The advantage of the curve analysis method is that it is easy to calculate. Model-based measurement parameters are complex, but they provide more specific information about vascular physiology [31]. In this study, we compared perfusion parameters between benign lesions and malignant lesions. TTP was significantly different. TTP is the time that contrast enhancement reaches the peak. A shorter TTP indicates the shorter time needed to reach the peak. Then it can be explained that the blood vessels are more abundant in the corresponding lesions. We further divided the lesions into two subgroups. The lesions were divided into two groups in the benign lesion group according to SUVmax ≤ 3.0 and SUVmax > 3.0. All perfusion parameters did not show obvious differences between SUVmax ≤ 3.0 and SUVmax > 3.0. This indicates that SUVmax does not affect the perfusion parameters in benign lesions. In the malignant lesions group, the same results were found.

Moreover, we determined the correlations between perfusion parameters to understand further the physiological significance of semi-quantitative parameters in intraprostatic lesions. They may reflect gross angiogenesis within a focal lesion. AT is arrival time, the point in time when contrast enhancement starts. A shorter AT indicates that the contrast agent flows into the lesion in a shorter time. It further shows that the lesion is rich in blood vessels. W-in is the fitted line’s slope between AT and the end of wash-in, reflecting the speed at which the contrast agent flows into the lesion. The slope is significantly correlated with blood flow so that it can be used to evaluate perfusion within a lesion. Another parameter, iAUC, is the initial area under the curve in 60 s. It suggests that iAUC denotes a combination of blood flow and permeability. These correlations may help select the most suitable semi-quantitative parameter to represent tumor perfusion, flow, and angiogenesis in daily practice.

Angiogenesis is an essential process in tumor growth, and a multitude of pharmacologic therapies primarily target angiogenesis by affecting vascular endothelial growth factor (VEGF) ligand binding [32]. Microvascular distribution is considered a vital sign of neovascularization, responsible for local growth and tumor metastasis [33,34]. In these applications, the potential of PSMA as an imaging biomarker is related to the exact function of PSMA in tumor-related endothelium. Conway et al. [35] demonstrated that PSMA is required for angiogenesis in vivo and is essential for endothelial cell invasion in vitro. Their results of linking PSMA with p21-activated kinase regulation suggest that PSMA is an important regulator of endothelial cell invasion and angiogenesis and may be a therapeutic target for angiogenesis-related diseases. Chang et al. [36] proved that PSMA was consistently expressed in the neovasculature of a wide variety of malignant neoplasms. More malignant lesions usually have more abundant microvascular structures [37,38,39,40]. DCE-MRI is one of the most mature imaging biomarkers of tumor microvessels. DCE-MRI and PSMA-PET can be performed simultaneously in a PET/MRI scanner so that these markers can be directly correlated.

Early research indicated that ADT causes a reduction of blood flow in the prostate gland that precedes apoptosis of the epithelium [41,42]. Another study held a different opinion. Roe et al. [43] reported that their key findings were the increased tumor vascularization following ADT. However, the above experimental conclusions are based on animal experiments. Human studies characterizing vascular effects following ADT are limited. The long-term impact of ADT on tumor vascularization needs to be further investigated. The effects following radiotherapy of prostate measured with quantitative MRI were reported by Kershaw et al. [44] They proved that tumor blood flow decreased after treatment. Nevertheless, because of this study’s relatively small sample size, a larger number of sample systematic quantitative studies are needed. Therefore, the effect of radiotherapy on the microvascular structure of the human prostate has not been conclusively established. Based on what has been discussed above, ADT and radiotherapy’s effects on microvasculature were not considered in this study. There are some limitations to our study. The patient cohort is relatively small. There are only eleven patients who performed radical prostatectomy (RP) after scanning. Therefore, we were not able to take histopathology results as a gold standard.

## 4. Materials and Methods

### 4.1. Patients

This retrospective study was approved by the institutional ethics review board (EA1/060/16), and informed consent was waived for retrospective analysis.

**Figure 4:**
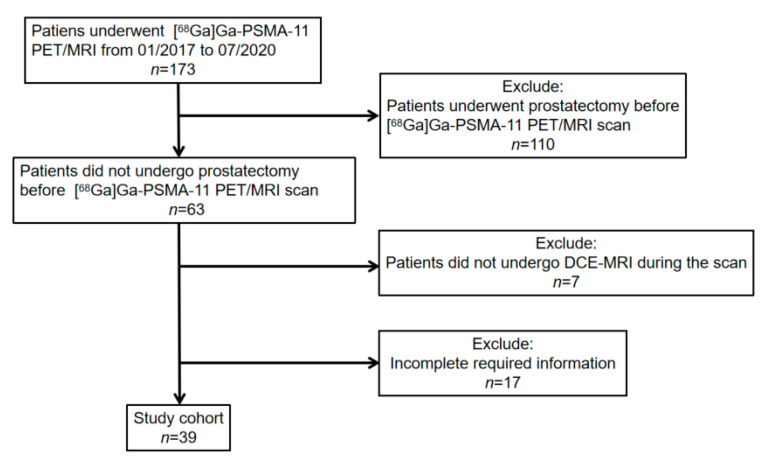
[^68^Ga]Ga-PSMA-11 PET: gallium 68-labeled prostate-specific membrane antigen PET, DCE-MRI: Dynamic contrast-enhanced magnetic resonance imaging.

We enrolled and excluded patients by the following criteria. Inclusion criteria: (1) patients with biopsy-proven prostate cancer who underwent [^68^Ga]Ga-PSMA-11 PET/MRI between January 2017 and July 2020 in our clinic; (2) necessary information could be obtained. Exclusion criteria: (1) patients, who underwent radical prostatectomy before scanning, were excluded; (2) patients did not undergo DCE-MRI during the scan. Patients’ inclusion and exclusion are summarized in the flowchart in Figure 4.

Patients who did not undergo radical prostatectomy before [^68^Ga]Ga-PSMA-11 PET/MRI and performed DCE-MRI in [^68^Ga]Ga-PSMA-11 PET/MRI examination were retrospectively selected from our institute’s database and included in this analysis. All patients were confirmed PCa by systematic biopsy before [^68^Ga]Ga-PSMA-11 PET PET/MRI scanning. Biopsy techniques included 12-core prostate biopsy and 14-core prostate biopsy. The needle biopsy technique introduced by Hodge et al. [45] has become the gold standard method for diagnosing prostate cancers. Both 12-core and 14-core prostate biopsy significantly increase the detection rate of prostate cancer and the accuracy of the biopsy Gleason score [46,47]. Clinical characteristics are compiled in Table 5.

### 4.2. [^68^Ga]Ga-PSMA-11 PET/MRI Imaging Protocol

[^68^Ga]Ga-PSMA-11 was synthesized using a clinical-grade ^68^Ge/^68^Ga radionuclide generator (Eckert & Ziegler Radiopharma GmbH, Berlin, Germany) and PSMA-HBED-CC (ABX GmbH, Radeberg, Germany). Patients were imaged after 79 (72, 109) min after intravenous injection of a mean activity of 162.2 ± 22.1 MBq (4.4 ± 0.6 mCi) [^68^Ga]Ga-PSMA-11, corresponding to activity: 1.8–2.2 MBq (0.049–0.060mCi) per kilogram bodyweight. To minimize the halo artifact caused by scattering overcorrection associated with high renal and urinary tracer activity, furosemide is injected 30 min before the start of PET acquisition. Patients were asked to void urine immediately before the start of the examination. No adverse effects were observed after [^68^Ga]Ga-PSMA-11 injection.

PET and MRI were performed using the same protocol for every patient. Imaging was obtained with a 3.0 T PET/MRI system (SIEMENS MAGNETOM Biograph mMR, Erlangen, Germany). The acquisition was split into two parts. First, body PET/MRI cover from the vertex to mid-thigh was performed with 3 min of PET acquisition in each bed position, with coverage of 24 cm. Pre-contrast MRI sequences were acquired simultaneously using a combination of a dedicated mMR head-and-neck coil and phased-array mMR body surface coils. Siemens StarVIBE eliminates motion artifacts. The second part was a dedicated MRI scan of the pelvis, followed by PET data reconstruction. MRI sequence parameters are summarized in Table 6. Reconstruction was conducted with an ordered subset expectation maximization algorithm (OSEM), with 3 iterations/21 subsets, based on an x-matrix acquisition with a 4 mm Gaussian filter and relative scatter scaling. Contrast-enhanced agent gadobutrol (Gadovist^®^, Bayer Pharma AG, Berlin, Germany) is intravenously administered at a clinical dose of 0.1 mmol/kg bodyweight. Following the acquisition of precontrast data, a total of 60 contrast-enhanced data were obtained, with the start of the first postcontrast acquisition corresponding with the start of the contrast injection.

### 4.3. Image Analysis

Post-processing of all imaging data was performed with a dedicated post-processing software, Syngo.via (Siemens Healthcare, Erlangen, Germany). Regions of interest (ROIs) were defined as regions with an abnormal signal on MRI images and manually drawn. The MRI datasets were interpreted using PI-RADS version 2.1 [48]. Lesions with PI-RADS 1 to 3 were considered benign, while those with PI-RADS 4 and 5 were considered malignant. SUVmax is measured based on ROI, which is defined as corresponding to the finding on T2-weighted imaging (T2WI). All images were read by the same double-trained doctor. All perfusion parameters extracted from time-intensity curves were generated using Syngo.via MR Tissue 4D (Siemens Healthcare; Erlangen, Germany). Perfusion parameters, including arrival time (AT), time to peak (TTP), wash-in slope (W-in), wash-out slope (W-out), peak enhancement intensity (PEI), and initial area under the 60-sec curve (iAUC), were calculated. For the time-intensity curve, X-axis refers to time, and Y-axis refers to the ratio between baseline and post-contrast intensity. Previous publications suggested values between SUVmax 2.0 to 3.0 as appropriate cutoff values to minimize false-positive interpretation of faintly PSMA positive uptake [49,50]. A cutoff of SUVmax 3.0 was selected in this study. The definition of the above parameters is presented in Table 7.

### 4.4. Statistical Analysis

The DCE-MRI parameters between benign and malignant lesion groups were compared by Mann–Whitney U test. Comparison of parameters between benign lesions with SUVmax ≤ 3.0 and SUVmax > 3.0 was accessed by Mann–Whitney U test, as were malignant lesions. Pearson correlation was used to determine the correlations among various DCE-MRI parameters. All statistical analyses were performed with statistical software SPSS 25 for Windows (IBM Corp, Armonk, NY, USA). The significance level was set to two-tailed *p* < 0.05. Patient demographics and clinical characteristics are summarized using descriptive statistics. Normal distributed data are reported as mean ± standard deviation (SD), and non-normal distributed data are reported as median (interquartile range) (IQR Q1, Q3).

## 5. Conclusions

In conclusion, various DCE-MRI parameters can be used to quantify perfusion in intraprostatic lesions. DCE-MRI parameters reflect the perfusion characteristics of intraprostatic lesions with malignant lesions demonstrating significantly shorter TTP. There is a moderate to strong correlation between DCE-MRI parameters. Semi-quantitative analysis reflects how malignant lesions show significantly higher SUVmax than benign lesions.

## Figures and Tables

**Table 1:** Comparison of parameters between benign and malignant lesions.

Parameter	Benign Lesions			Malignant Lesions			
	Median	Q1	Q3	Median	Q1	Q3	*p* Value
SUVmax	2.3	1.5	3.7	7.0	4.2	11.5	*p <* 0.05 *
AT(min)	0.47	0.40	0.57	0.47	0.39	0.56	*p* > 0.05
TTP(min)	1.09	0.84	1.32	0.95	0.75	1.22	*p <* 0.05 *
W-in	0.13	0.07	0.18	0.12	0.08	0.22	*p* >0.05
W-out	0.01	0.01	0.02	0.01	0.01	0.02	*p* >0.05
PEI	0.21	0.15	0.28	0.20	0.15	0.26	*p* > 0.05
iAUC	0.08	0.05	0.11	0.08	0.05	0.12	*p* > 0.05

AT: arrival time, TTP: time to peak, W-in: wash-in slope, W-out: wash-out slope, PEI: peak enhancement intensity, iAUC: initial area under the 60-sec curve, SUVmax: standardized uptake values maximum, * *p* < 0.05.

**Table 2:** Comparison of parameters between benign lesions with SUVmax ≤ 3.0 and SUVmax > 3.0.

Parameter	SUVmax ≤ 3.0			SUVmax > 3.0			
	Median	Q1	Q3	Median	Q1	Q3	*p* Value
SUVmax	1.6	1.2	2.3	4.7	3.6	6.2	*p <* 0.05 *
AT(min)	0.47	0.39	0.58	0.47	0.44	0.56	*p* > 0.05
TTP(min)	1.13	0.92	1.35	0.92	0.78	1.24	*p* > 0.05
W-in	0.13	0.07	0.18	0.13	0.08	0.21	*p* > 0.05
W-out	0.01	0.01	0.02	0.01	0.01	0.02	*p* >0.05
PEI	0.21	0.16	0.28	0.21	0.12	0.27	*p* > 0.05
iAUC	0.09	0.05	0.11	0.07	0.05	0.12	*p* > 0.05

AT: arrival time, TTP: time to peak, W-in: wash-in slope, W-out: wash-out slope, PEI: peak enhancement intensity, iAUC: initial area under the 60-sec curve, SUVmax: standardized uptake values maximum, * *p <* 0.05.

**Table 3:** Comparison of parameters between malignant lesions with SUVmax ≤ 3.0 and SUVmax > 3.0.

Parameter	SUVmax ≤ 3.0			SUVmax > 3.0			
	Median	Q1	Q3	Median	Q1	Q3	*p* Value
SUVmax	2.0	1.0	2.2	8.2	5.5	12.2	*p <* 0.05 *
AT(min)	0.49	0.47	0.98	0.47	0.39	0.55	*p* > 0.05
TTP(min)	0.95	0.66	1.12	0.96	0.77	1.22	*p* > 0.05
W-in	0.16	0.08	0.28	0.12	0.08	0.22	*p* > 0.05
W-out	0.01	−0.003	0.02	0.01	0.01	0.02	*p* > 0.05
PEI	0.20	0.14	0.32	0.21	0.15	0.25	*p* > 0.05
iAUC	0.08	0.05	0.19	0.08	0.05	0.12	*p* > 0.05

AT: arrival time, TTP: time to peak, W-in: wash-in slope, W-out: wash-out slope, PEI: peak enhancement intensity, iAUC: initial area under the 60-sec curve, SUVmax: standardized uptake values maximum, * *p <* 0.05.

**Table 4:** Pearson correlation analysis between the perfusion parameters.

	AT	TTP	W-in	W-out	PEI	iAUC
AT	1	−0.17 *	0.18 *	−0.05	−0.004	0.18 *
TTP	-	1	−0.45 **	0.71 **	0.17 *	−0.31 **
W-in	-	-	1	−0.30 **	0.57 **	0.95 **
W-out	-	-	-	1	0.41 **	−0.18 *
PEI	-	-	-	-	1	0.70 **
iAUC	-	-	-	-	-	1

Data are Pearson correlation coefficient. AT: arrival time, TTP: time to peak, W-in: wash-in slope, W-out: wash-out slope, PEI: peak enhancement intensity, iAUC: initial area under the 60-sec curve, * *p <* 0.05, ** *p <* 0.01.

**Table 5:** Summary of clinical characteristics.

Characteristics	*N* = 39
Age at scan (years)	69 ± 9
PSA (ng/ml) at scan time	8.70(5.18, 18.83)
Biopsy Gleason score (n)	
3 + 3	8
3 + 4	8
4 + 3	8
4 + 4	7
4 + 5	2
5 + 4	3
5 + 5	3
Treatment	
ADT prior to scan (n)	2
ADT ongoing at the time of scan (n)	3
Radiotherapy prior to scan (n)	3

ADT: Androgen deprivation therapy.

**Table 6:** Imaging parameters used for MRI.

Sequence	TR/TE (msec)	FOV (mm)	Flip Angle (Degrees)	Section Thickness (mm)	Voxel Size (mm)
T2WI HASTEAxial	1400.0/95.0	400	160	5.0	1.3 × 1.3 × 5.0
T1WI FS VIBE	1600.0/96.0	350	160	4.0	1.1 × 1.1 × 4.0
T2WI Axial	5500.0/103.0	180	150	3.0	0.5 × 0.5 × 3.0
T2WI Sagittal	1600.0/96.0	350	160	4.0	1.1 × 1.1 × 4.0
T2WI Coronal	4500.0/102.0	200	173	3.0	0.4 × 0.4 × 3.0
DWI	11,600.0/70.0	280		3.0	2.5 × 2.5 × 3.0
T1WI FS TWIST dynamic	7.41/3.30	260	12	3.5	1.4 × 1.4 × 3.5
T1WI STARVIBE	3.71/1.77	360	9	1.2	1.1 × 1.1 × 1.2

**Table 7:** Definition of DCE-MRI Parameters.

Parameter	Definition
AT	arrival time: point in time when contrast enhancement starts
TTP	time to peak: time from arrival time to end of wash-in
W-in	wash-in: slope of the fitted line between AT and end of wash-in
W-out	wash-out: slope of the fitted line between start of wash-out and end of measurement
PEI	peak enhancement intensity: value of concentration when the contrast enhancement reaches the highest concentration
iAUC	initial area under curve in 60 s

## Data Availability

The datasets analyzed and generated during this study are included in this published study.

## Abbreviations

AT	Arrival time;
BPH	Benign prostatic hyperplasia;
CT	Computed tomography;
DCE-MRI	Dynamic contrast-enhanced magnetic resonance imaging;
DWI	Diffusion-weighted imaging;
FOV	Field of view;
Ga	Gallium;
GS	Gleason score;
iAUC	initial area under curve;
MRI	Magnetic resonance imaging;
mpMRI	Multiparametric magnetic resonance imaging;
PCa	Prostate cancer;
PEI	Peak enhancement intensity;
PET/CT	Positron emission tomography / computed tomography;
PET/MR	Positron emission tomography /magnetic resonance;
PI-RADS	Prostate Imaging Reporting and Data System;
PSA	Prostate-specific antigen;
PSMA	Prostate-specific membrane antigen;
ROI	Region of interest;
SUV	Standardized uptake value;
TE	Echo time;
TR	Repetition time;
TTP	Time to peak;
T2WI	T2-weighted imaging;
W-in	Wash-in slope;
W-out	Wash-out slope.
